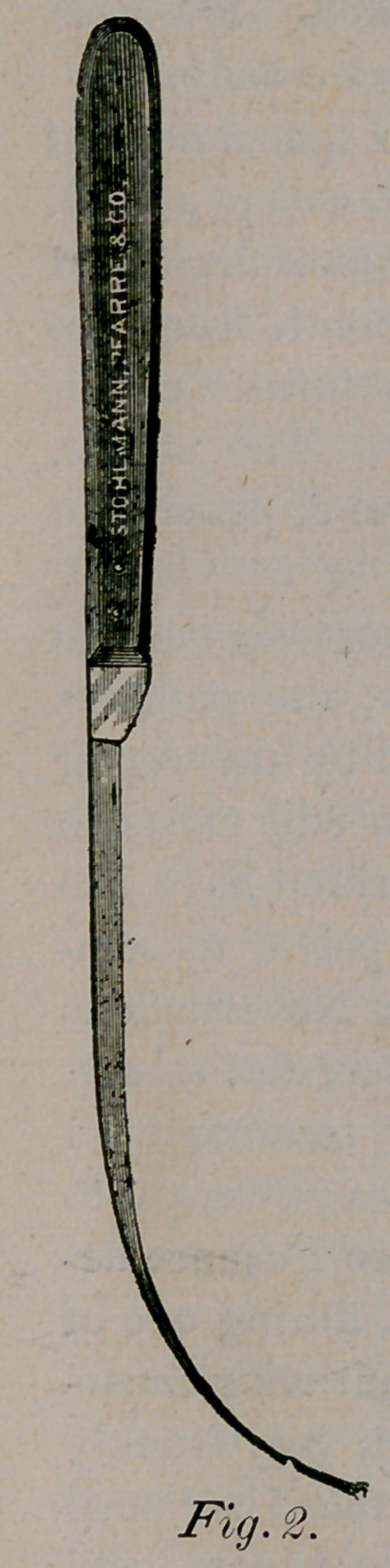# Report on Surgery for the Seventh Congressional District

**Published:** 1885-08

**Authors:** Everard H. Richardson

**Affiliations:** Member of the Section on Surgery, Cedartown, Ga.


### REPORT ON SURGERY FOR THE SEVENTH CON GRESSIONAL DISTRICT.

READ BEFORE THE MEDICAL ASSOCIATION OF GEORGIA AT ITS THIRTY-SIXTH ANNUAL SESSION, BY EVERARD H. RICHARDSON, M. D., MEMBER OF THE SECTION ON SURGERY, CEDARTOWN, GA.

### REMOVAL OF NECROSED FRAGMENT OF THE SKULL, RESULT OF GUN-SHOT WOUND, SIXTEEN YEARS AFTER RECEIPT OF INJURY.

*Case I.*—Mr. J. C., native of New Orleans, aged fifty years, machinist and engineer, during the year 1868, while on the frontiers of Kansas, received a gun-shot wound of left parietal bone, the ball striking about two inches in front of lambdoid suture. The missile, which was judged to be small, was fired from below the subject. It did not penetrate, but simply "grazed" the surface of the cranium, carrying off a small portion ot the external table of the parietal bone. Mr. C. did not fall from his horse, but was slightly stunned from the blow. He states that there was a considerable hemorrhage, which was checked by a surgeon without ligature, who saw him soon after the casualty and dressed his wound with lint and cold water. The wound healed entirely in three months, leaving a depression in skull. Two years later he began to suffer pain, and pus was discharged from the cicatrix of the old wound. During the ensuing eight years the wound alternately healed and discharged pus—patient during the intervals enjoying comparative immunity from serious suffering or inconvenience. At about this date (eight years from receipt of injury), he jumped from a locomotive while in rapid motion, striking first upon his head. From this time the wound of the head waxed worse, suppurating profusely, and was attended with great pain.

On the 26th day of October, 1884, Mr. C. presented himself, narrating substantially the above history. His presence was almost intolerable on account of the *stench* of the freely flowing pus that issued from a number of sinuses burrowing beneath his scalp. With probe I readidly detected "dead bone," and introducing forceps I attempted its removal. This I could not do because separation of the sequestrum was not yet complete. One week later a piece of necrosed bone was removed without difficulty. It is herewith exhibited for your inspection. It is quite evident, I think, that both tables of the cranium are necrosed. The actual measurement of the fragment that has been removed is two and a half inches in length and two inches in width. The wound was carefully examined with my fingers and probe, and no remaining dead bone discovered. Carbolized compress, frequent irrigations with a one to forty solution of carbolic acid, comprised the after-treatment. Immediately after the removal of the fragment of bone, he boarded the cars and rode a distance of twenty-five miles. He expressed great relief from the operation and said that the sensation of pressure and "dead weight" was entirely removed from his head. One month after the operation, suppuration had almost entirely ceased and the wound had almost completely healed. What the outcome of this case will be I am unable to say. However, the patient is happy and says that he is a new man, and that he has not lost a day from his work since the removal of the dead bone. From the first I suspected an underlying syphilitic element in the case and ordered round doses of iodide of potassium combined with bichloride of mercury.

RADICAL CURE OF HYDROCELE BY INJECTION OF CARBOLIC ACID.

*Case II.*—Alfred McC., colored, aged forty-five years, farm laborer, came to me to be "tapped" for single hydrocele that had existed for ten years, stating that it had been his custom to have the fluid drawn off about twice during the year, which enabled him to get along with his work.

I informed him that I could give him permanent relief, and thus obviate the necessity for "tappings," by a very insignificant operation, if he would give his consent. He readily assented, provided I would not "lay him up." Accordingly I introduced a trocar and canula, and emptied the sac entirely. When this was accomplished, I introduced a long needle attached to a hypodermic syringe, which had been previously charged with thirty minims of Squibb's liquid carbolic acid, through the still remaining canula, held *in situ*, and injected the acid into the bottom of the sac, permitting it to remain permanently there after withdrawing the canula. No pain of consequence was complained of. This was on Sunday. He remained in his house till the following morning, when he left his room for the harvest field, and began work as a reaper. Five or six days after, on passing the field where he was engaged in harvesting, I called him to me and examined him. I found the scrotum and testicle considerably swollen, but he did not seem to mind it, and returned to the field, where he persisted in his work, making a full hand continuously without stopping so much as a day on account of the operation. Six months after this procedure, there had been no re-accumulation of fluid, and I exhibited the patient to Dr. C. H. Harris, who concurred with me that the relief was permanent. Dr. Levis, the able author and promulgator of this method of treating hydrocele, states in the Philadelphia *Medical Times*, of November, 1880, that he had used this procedure since 1872, and that he "had never seen suppuration or sloughing follow this manner of dealing with hydrocele."

During the winter of 1882, at Professor Robert Weir's clinic in the College of Physicians and Surgeons, New York City, I saw him quite frequently thus treat subjects of hydrocele, and generally with good results. This case recorded here is but an average type of several such treated by myself, after this method, that has been attended with the same uniform success. In no case coming under my personal observation has untoward symptoms supervened, after this simple method has been practiced, for the radical relief of hydrocele.

### COMPOUND COMMINUTED FRACTURE OF TIBIA AND FIBULA.

*Case III.*—February 4th, 1885, Robert Mackay, a colored convict, aged twenty-eight years, belonging to Penitentiary Camp No. 2, located in Polk county, while engaged in raising iron ore, was felled to the ground by the caving of a bank of dirt, and the falling of a large piece of iron ore, which struck his left leg about midway between the knee and ankle joints. I saw him shortly after the accident, and had him conveyed on the cars of the East and West Railroad to my hospital in Cedartown. By inspection, a compound comminuted fracture of the shaft of both bones of the leg was diagnosed. The tibia jutted through the flesh ; considerable hemorrhage had occurred.

The foot was markedly everted, and shortening existed. I decided to make an effort to save the limb by rendering the wound entirely aseptic, and hermetically sealing it after the bones had been perfectly coaptated. With a Davidson's syringe, the wound in all its ramifications was thoroughly irrigated with a five per cent. solution of carbolic acid. Assisted by two negro convicts, he was etherized, the wound covered by several layers of carbolized lint, and the entire limb, from knee to below mallioli, was thickly enveloped in absorbent cotton. The object of this was to make proper allowance for subsequent swelling, just as though I was applying the plaster of Paris dressing. After coaptating the broken ends of bones, two gutta percha splints, having been rendered perfectly flexible by steam, were accurately moulded to each side of the limb, from knee downwards, embracing entire length of limb. A fenestra was made in the splint immediately over the injury. This " window " was effectively occluded by carbolized absorbent cotton, and the splints were securely held in position by the roller bandage. An improvised side splint was adjusted to the outer side of the limb, extending from axilla to external malliolus, for the purpose of keeping the great-toe constantly on a line with the inner border of the patella. This constituted the dressing.

The patient was placed in bed, and was put on a diet of milk, eggs, meat and vegetables. He received no medication, except enough opium to keep him quiet and free from pain. I saw him twice daily and relied *solely* upon the thermometer as a danger signal to inform me of the condition of the reparative process. For the first few days the temperature remained from a degree to a degree and a half above normal. This was to be expected. I was willing to stand by and refrain from removing the dressing unless the temperature should have run up several degrees, pointing unmistakably to brewing mischief. For fifteen days the dressing was not disturbed. At this time I was curious enough to open the window in the splint over site of injury, and ascertained that the wound had not entirely healed, but not a drop of pus had formed, and the outlook seemed promising and eminently satisfactory. Oakum saturated with balsam of Peru was applied to the wound and changed twice daily. *En passant*, I will mention that for open, suppurating wounds, I know of no dressing equal to this one. I learned of its use from personal observation in Bellevue Hospital at the clinics of that deservedly celebrated surgeon, Lewis A. Sayre. After about a month the splints were removed and the limb was found all right. Passive motion of knee and ankle joint was instituted. Two months have now elapsed since the practice occurred there. No shortening, and the limb is in every respect as good as it was prior to receipt of the injury.

### FISTULA IN ANO TREATED WITH ELASTIC LIGATURE.

*Case IV.*—Warren B., colored, aged thirty-three years, was sent to me from Cherokee county, Ala., to treat for an abscess in anal region that had existed for two years. He was a scrofulous subject, and on examination I found that he had a deposition of tubercles in apex of left lung. For a year he had been in bed most of the time and was in a bad plight for an operation. On the 4th of March, aided by Drs. Liddell and England, he was etherized, and with the knife and director I laid open several sinuses, none of which were found to enter the gut. The main sinus extended some distance up along the course of the rectum, hugging close to the gut. The bottom of this was reached and also laid open from within outwards. Considerable hemorrhage followed, and I decided to pack tightly with lint and muriated tincture of iron and desisted from further use of the knife. On subsequent dressing, the wound was washed regularly with warm carbolized water and packed lightly with oakum and balsam of Peru. After one month I decided that the reparative process would never be accomplished without a division of the sphincter ani muscles, and owing to the condition of the patient, and the sinus extending so high up, I deemed the further use of the knife too hazardous, and I concluded to operate with the elastic ligature. In harmony with this conclusion, assisted by Dr. C. H. Harris, the patient was etherized, and with Helmuth's modification of Allinham's ligature carrier (Fig. 1,) which is here shown, I passed the point, armed with a stout, solid rubber ligature to the bottom, of the upper portion of the sinus from without inwards and pushed the concealed blade into the rectum, bringing the ligature out at the anus. (The author of this ingenious instrument intended that it should be introduced from within the rectum outwards.) Both ends of the ligature were threaded through a leaden ring and drawn taut, when the ring was clamped by a stout pair of forceps. It was thirteen days before the ligature cut through the tissues and sphincter muscles, and the poor patient suffered excruciating pain, requiring large doses of morphia to keep his condition at all bearable. I, however, think that I failed to draw the ligature as tightly as I would again do. This may account, perhaps, for the violence of the pain from which my patient suffered. Allinham, after using the elastic ligature in several hundred cases, says: "The ligature cuts through in six days. In simple cases there is little or no pain inflicted by the operation, and the patient can walk about without danger."

*Fig 1.*

It was about two months before the sphincters united and he gained control of movements from his bowels. The bowels were confined for first week after application of ligature. The fistula finally healed, and I was congratulating myself upon the ultimate results, when I discovered an abscess forming in the opposite ischio-rectal region.

Upon considering my patient's depraved physical condition, his bad hygienic surroundings, etc., I decided that further operative interference would be unjustifiable, and I abandoned his case with the sad reflection that the resources at my command were impotent and powerless to mitigate the sufferings or avert the death that awaited the patient. Nevertheless, I think well of the elastic ligature in suitable cases, and shall again use it when an opportunity is offered.

*Fig. 2.*

In this connection I show an admirable curved bistoury (Fig. 2,) with a probe attached, which dispenses with the director, and in the simpler cases of fistula in ano subserves a most excellent purpose.

### EMMETT'S OPERATION FOR LACERATED CERVIX UTERI.

*Case V.*—Mrs. A., aged thirty years, widow, and a native of Georgia, has been a sufferer from uterine disease for five years. She describes the usual pelvic and back pains, profuse lucorrhœa and menorrhagia. Locomotion is attended with such pain that she has desisted from all effort to walk, and remains in a recumbent posture all the while. She is, in fact, bed-ridden, and for two years has constantly indulged in the opium habit. Insomnia is the bane of her life, and she has great repugnance for food. She is the most miserable of human creatures, being a confirmed hypochondriac, and it seems to be a positive luxury for her to contemplate the "dark side" of the future. Digital examination reveals a very sensitive, enlarged and retroverted uterus, with an extensive, unilateral lacerated cervix. The whole texture of cervix is implicated, and the laceration extends to the utero-vaginal junction. With Sims's speculum introduced, the cervix is observed to be intensely engorged, presenting the appearance of a piece of raw beef. Hyperplasia and eversion of cervix is marked, and the parts are bathed in a profuse leucorrheal discharge.

About one month was consumed in preparing the patient for the radical cure of the lacerated cervix. The hot douche was used regularly. Applications of glycerine, alum, tannin, iodine, carbolic acid, etc., were in turn used. Finally, assisted by Drs. Harris and Liddell, with the patient etherized and placed in Sims's position, I pulled the uterus down with tenaculum, and after thorough denudation with Emmett's scissors, four silver sutures were introduced, and the raw surfaces nicely coaptated. She was placed in bed, and the vaginal douche was used twice daily.

On the 10th day she was examined with Sims's speculum and all the sutures removed. Union was found perfect, except a small portion in the angle of the wound. A few applications of nitric acid caused union to take place in this spot, and one month from the date of operation the uterus bore no likeness to its former condition, so great had been the change for the better. The uterus, though freely movable, would not tolerate any form of pessary to keep it in a more anteverted position.

Albert Smith's and Cutter's pessaries failed most signally to benefit this patient in the least degree. Clearly, the patient's physical condition has been very greatly improved by the repair of the laceration, notwithstanding the retroversion still exists in about the second degree. Her appetite and nutrition have improved greatly. She is comparatively free from pelvic discomfort, and walks around and rides out occasionally. Nevertheless, she seems to *luxuriate* in saying that she is no better, and stoutly declares that she has received no benefit from my treatment. I begin to suspect that her reason is dethroned. I am powerless to break up the opium habit with her—why?—because I cannot deprive her of her personal liberty. About this time, during one of my visits, with her eyes riveted upon me and radiant with unnatural brightness, she, with the dignity of a queen, very frankly said to me that I had not been trying to cure her; that I had been visiting her for the purpose of purloining valuable papers, and that, in collusion with a near relative of hers, I had robbed her of a large sum of money! The truth dawned upon me that this poor creature was insane. She was adjudged a lunatic and sent to an insane asylum. She was kept there about four months, and while there received no local treatment for the uterine trouble. At the expiration of this time she returned home, relieved entirely of her mental trouble and the opium habit.

On her return I visited her, and found her mind clear in every respect. I made no allusion to her trouble, except to inquire concerning the treatment she had received, and to know if she had had any uterine discomfort. I soon ascertained that the uterus was discharging all of its functions naturally and painlessly.

One year has now elapsed since this good lady returned from the asylum. Her health has been perfect, and to-day she is as happy as the birds that surround her rural home. I am aware that the history of this case presents no novel features of interest, and may be considered "common place." It is introduced here for two reasons, viz.:

1st. To show the really brilliant results that ensue from Emmett's operation of repairing the lacerated cervix. I fear that Southern physicians, with our British brethren, do not fully appreciate the pathological significance of this condition of the uterus as an etiological factor in the production of uterine disease. Emmett's operation to remedy this condition and its consequences is a real advance in our art to relieve the ills of suffering woman, and is one of the most brilliant triumphs of modern gynecology.

2d. I desire to express my endorsement of the views of that clear-headed practitioner and courtly gentleman, Fordyce Barker. I agree with him perfectly that this organ has no fixed mathematical position in the pelvis. Every uterus is a "law unto itself," and it may vary in its position with different individuals without producing disease. I confidently assert that the position of the uterus here described is not a bar to conception, or successfully carrying the same to full term of gestation. In the majority of instances, to allow a woman who is a victim of "womb disease" to using a pessary *condemns* her to wear it the balance of her life, and it engenders a condition of mental erethism, which renders her miserable without one. The conviction is fastened upon the minds of these poor creatures that it would be impossible for them to live without being the possessors of one of these humane (?) contrivances.

## Figures and Tables

**Fig. 1. f1:**
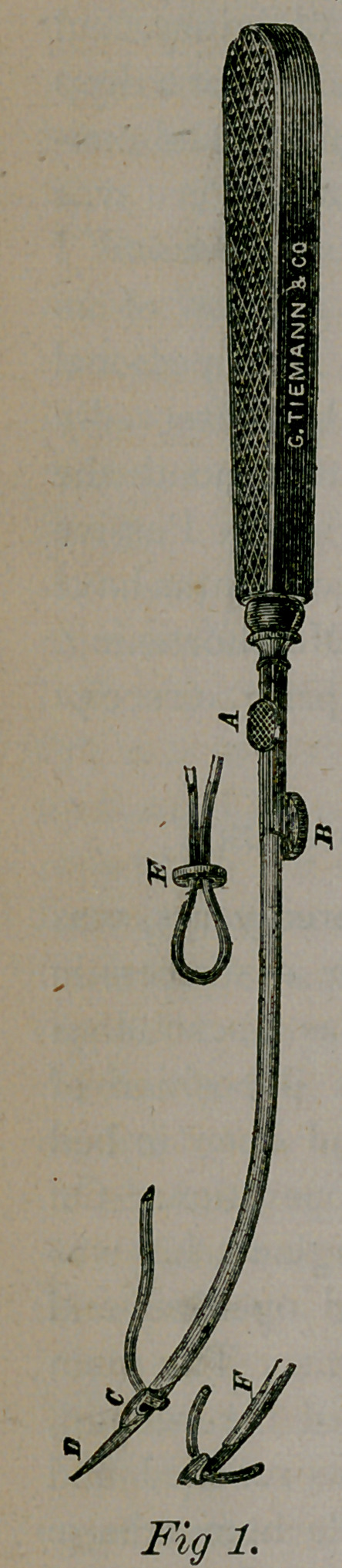


**Fig. 2. f2:**